# Proton Pump Inhibitors in Pediatric Gastroesophageal Reflux Disease: A Systematic Review of Randomized Controlled Trials

**DOI:** 10.3390/children11030296

**Published:** 2024-03-01

**Authors:** Sara María Fernández-González, Ana Moreno-Álvarez, Alfonso Solar-Boga

**Affiliations:** Pediatric Gastroenterology, Hepatology and Nutrition Unit, Department of Pediatrics, A Coruña University Hospital, Area Sanitaria A Coruña-Cee, 15006 A Coruña, Spain; sara.maria.fernandez.gonzalez@sergas.es (S.M.F.-G.); alfonso.solar.boga@sergas.es (A.S.-B.)

**Keywords:** gastroesophageal reflux disease, efficacy, safety, proton pump inhibitors, pediatric patients, systematic review

## Abstract

This systematic review was conducted with the objective of understanding the efficacy and safety of proton pump inhibitors (PPIs) in the pediatric population. We used PubMed to identify randomized controlled trials (RCTs) published between 1 June 2010 and 30 June 2023, performed in patients from birth to 18 years old with gastroesophageal reflux disease (GERD) who received treatment with any PPI. This literature search yielded 76 articles and 13 of these met the inclusion criteria. For infants, PPIs were equal to placebos in reducing GERD symptoms in four articles. In one article, the numbers of GER episodes and esophageal acid exposures were lower in infants who received PPIs in the left lateral position, but there was generally no significant improvement in symptoms. In another publication, the combination of PPIs and feeding modifications (FMs) was not more effective than PPIs alone. For children and adolescents, PPIs were effective in improving symptoms and achieving endoscopic healing, which was subsequently maintained. To conclude, PPIs are not effective in reducing the symptoms related to GERD in infants but are effective in older children, where histological remission can be seen. Generally, PPIs are well tolerated, but it is important to remember the possible adverse events (AEs), especially if PPIs are used for an extended period.

## 1. Introduction

In the pediatric population, gastroesophageal reflux (GER) is very common, affecting approximately 50% of infants under three months old [1]. It consists of a physiological process and tends to resolve spontaneously at 12–24 months [2]. Although most episodes are asymptomatic and do not cause complications, on fewer occasions, they can cause troublesome symptoms, damage the esophagus, and/or affect the general condition of the child, thus constituting gastroesophageal reflux disease (GERD) [2,3].

The prevalence of GERD is difficult to establish because of the heterogeneity of the diagnostic criteria used in different studies [3,4,5]. The disease could occur in 25.5% of babies aged 0–1 month, decreasing to 1.1–1.6% by 1 year of age [3,5] and ranging from 0.9% to 18.8% [5,6] in childhood and adolescence. Higher rates of GERD are seen in children with a history of prematurity [7,8], developmental and neuromuscular disorders [9], a cow’s milk protein allergy [3,10,11,12], or pulmonary disease [13].

The clinical manifestations of GERD vary greatly depending on age. In infants, it is important to differentiate healthy infants with GER, frequently called ‘happy spitters’ [14] from infants with GERD. In older children and adolescents, symptoms similar to those in adulthood are more typical [14].

The management of GERD depends on age, clinical manifestations, and complications. Different guidelines for its diagnosis and treatment have been published over the last decade [11,14,15,16,17] with variable methodological approaches [18], but most share in common conservative initial management, especially in infants. When these aforementioned interventions are not sufficient, pharmacological treatment should be pursued. 

Proton pump inhibitors (PPIs), like omeprazole, lansoprazole, esomeprazole, rabeprazole, and pantoprazole, are the most well-known and used drugs for the treatment of GERD. They irreversibly block the gastric H,K-ATPase, inhibiting gastric acid secretions [19]. Although GERD guidelines indicate the use of PPIs only for the treatment of EE [11,20], they are often prescribed empirically for childhood reflux and other GERD-related symptoms without confirmation [21]. This has led to an increase in the prescription of these drugs for those of pediatric age in recent years [22,23,24]. 

PPIs are considered safe drugs, with most of the reported adverse events (AEs) being mild and related to digestive symptoms or skin reactions [14,21]. However, their chronic use has been linked to gastrointestinal [25] and lower respiratory tract infections [26]. In addition, some data suggest a possible increased risk of fractures through changing osteoclast activity [27,28] or the development of allergic disease as a consequence of chronic hypochlorhydria [21,29], especially when used in the first months of life or for an extended period. 

A systematic review on the efficacy and safety of PPIs in the treatment of GERD in children was previously published [30]. Since then, new treatment guidelines have emerged [18] and the use of PPIs has expanded. Therefore, our objective was to conduct a systematic review to understand the efficacy and safety of PPIs for GERD in pediatric patients.

## 2. Materials and Methods

This review was conducted in accordance with the guidelines outlined in the Preferred Reporting Items for a Systematic Review and Meta-Analysis (PRISMA) [31]. To structure the review process, the population, intervention, comparison, and outcome (PICO) model was followed [32,33] (see Table 1). Additionally, before starting the systematic review, the study was registered with the International Prospective Register of Systematic Reviews (PROSPERO) with the ID CRD42023473192.

### 2.1. Search Strategy

A systematic literature review was conducted to identify relevant publications on the efficacy of PPIs as a treatment for GERD in children. For this purpose, the main database MEDLINE (Pubmed) was searched. The search strategy used in the MEDLINE database was restricted to randomized controlled trials (RCTs) published between June 2010 and June 2023 because the previous systematic review included articles up until May 2010 [30]. 

The search was performed by two researchers independently in the PubMed database using the MESH terms filters: randomized controlled trial; child: birth–18 years; from 1 June 2010 to 30 June 2023. No language restriction was applied. 

The search formula used in the MEDLINE database was as follows: [“Omeprazole”[Mesh]] OR “Esomeprazole”[Majr:NoExp] OR “Lansoprazole”[Mesh] OR “Pantoprazole”[Mesh] OR “Dexlansoprazole” OR “Rabeprazol” OR [“Proton Pump Inhibitors”[Mesh]] AND [“Gastroesophageal Reflux”[Mesh] OR “Gastroesophageal Reflux”[Majr:NoExp] OR “Esophagitis, Peptic”[Majr:NoExp]] AND [“Infant”[Mesh] OR “Child”[Mesh] OR “Adolescent”[Mesh]].

### 2.2. Inclusion and Exclusion Criteria

The inclusion criteria were as follows:

1. The study was an RCT.
2. The target population was any pediatric patient (0 to 18 years of age) with GERD not secondary to another gastrointestinal pathology and receiving treatment with omeprazole, esomeprazole, lansoprazole, pantoprazole, rabeprazole, or dexlansoprazole.
3. One of the aims of the study was to evaluate the efficacy, AEs, and/or safety of PPI therapy.
4. The intervention consisted of a PPI and was compared with another PPI, another PPI dose, placebo, no treatment, or alternative treatment.
5. The outcome measure was the effectiveness and/or safety of the treatment for GERD in the pediatric population.

Studies with pathologies that can worsen GER or can confuse clinical manifestations, such as asthma, neurological disorders, cystic fibrosis, eosinophilic esophagitis, or gastroesophageal interventions, were excluded. 

### 2.3. Selection of Studies and Data Extraction

Two authors (S.F.G. and A.M.A.) independently selected the articles to be included in this review by searching the databases. They independently screened all titles and abstracts of identified RCTs for eligibility. If disagreement between the two reviewers transpired, a consensus was formed or a third reviewer (A.S.B.) acted as a referee. Duplicate references were eliminated. All studies deemed potentially relevant and those for which abstracts did not offer adequate information for inclusion or exclusion were retrieved in their entirety as full articles.

The total number of the sample and each group was analyzed for each study; the variables and characteristics of each treatment, the duration of the intervention, the follow-up time, and the results of each trial were recorded. 

Structured data extraction was performed from the original reports by 2 reviewers (S.MG. and A.M.A.) independently. The following data were extracted from the selected articles: study details (author, year of study, country, study design, sample size, duration of follow-up), participant details (number, age, sex, method of GERD assessment), PPI studied, type of intervention (non-pharmacological treatment: head and body position after meals or diet without cow’s milk proteins, or pharmacological treatments), control group (another PPI, placebo, or another treatment), treatment doses and treatment duration, primary and secondary objectives, outcome measures, treatment monitoring (pH monitoring, pH-MII, endoscopy, histological study), results, and number and type of AEs.

## 3. Results

### 3.1. Study Selection

**Figure 1:**
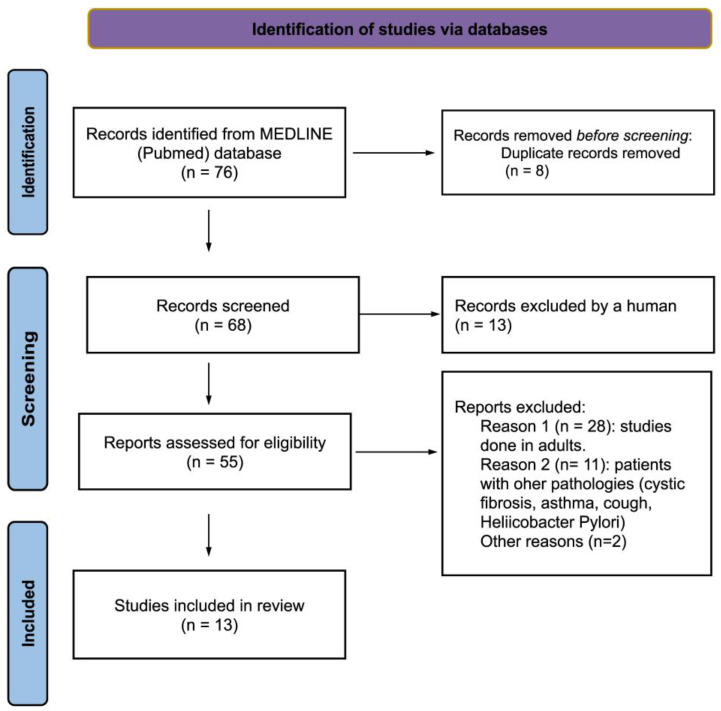
Flowchart of the search strategy and results based on the PRISMA statement [31].

The PubMed database search yielded 76 records. These were selected by title and reviewed by abstract, and 55 articles were analyzed for full-text review. Finally, 13 articles [34,35,36,37,38,39,40,41,42,43,44,45,46] that matched the selection criteria were selected. The results of the search are summarized according to the PRISMA statement 2020 in the flowchart in Figure 1. The reasons for the exclusion of articles that were read in full but not included in the review are summarized in Supplementary Table S1.

All the articles were published in 2010 or later. A total of 53.8% of the articles (7/1) were published by authors from the United States (US), 23.1% of the authors were from Poland (3/13), one was from Iran, one was from Australia, and one was from the Netherlands; most of the articles 84.6% (11/13) were multicenter studies that included European countries, the U.S., and Canada.

Data from 1166 participants (54.5% males) between 0 and 18 years old were included. The median sample size was 64 (ranging between 49 and 268). The age ranges in the studies varied widely. Six of them included patients under 1 year of age [34,35,38,39,40,41]. The remaining seven articles included patients older than 1 year: three included patients between 1 and 11 years old [44,45,46], one between 1 and 3 years old [43], one between 1 and 5 years old [42], one between 7 months and 18 years old [37], and one between 12 and 17 years old [36]. 

The studies were categorized according to the age groups of the participants (infants, children, and adolescents) due to the distinct manifestations of GERD symptoms in these populations and the potential variations in efficacy. 

For inclusion in this study, GERD was diagnosed in five studies by clinical criteria, in two studies by pH impedance, and the rest by clinical criteria were confirmed by endoscopic findings [36,42,43,44,45,46]. 

Esomeprazole was the most studied PPI in 5 of the 13 articles [34,39,41,43,44], while rabeprazole was examined in 3 articles [38,45,46], omeprazole in 2 [35,37], pantoprazole in 2 [40,42], and dexlansoprazole in 1 [36]. Five studies [38.46%] compared PPIs with placebo [34,36,38,40,41], another five [38.46%] were dose efficacy studies [42,43,44,45,46], 15.38% compared the use of PPIs with postural and dietary measures [35,39], and one study compared omeprazole with quince syrup [37]. 

Rescue medication was allowed in eight studies (non-bismuth-containing liquid antiacid or similar) [36,40,41,42,43,44,45,46]; one article specified that it did not allow rescue medication [38]; and no information in this regard was available for the rest of the studies.

### 3.2. Effectiveness of PPI

#### 3.2.1. Infant Population

We have identified six articles on children aged between 28 weeks postmenstrual age and 12 months with GERD [34,35,38,39,40,41]. These are summarized in Table 2. Two of these articles [34,35] studied neonates, one looked at patients from birth to 6 months of age [39], and the rest studied patients between 1 and 12 months.

In total, 101 neonates (45.7% male) were included [34,35] with postmenstrual age (PMA) between 28 and 60 weeks. The diagnoses were made according to clinical symptoms detected by video monitoring [34] or were pH impedance-associated [35]. 

Regarding effectiveness, Davidson et al. [34] compared esomeprazole with a placebo and found no significant differences in the number of GERD-related clinical manifestations. Jadcherla et al. [35] studied the impact of feeding strategies with acid suppression on esophageal reflexes. They compared neonates who received omeprazole with patients who also had feeding modifications (FM) (restrictive feeding strategies with enteral nutrition until 140 mL/kg/day, as well as body positional modifications). They concluded that PPI treatment with FM did not significantly improve esophageal reflexes, signs, or symptoms.

We retrieved four studies carried out on infants over 1 month old [38,39,40,41]. The studies included 523 infants (60.5% males) with a mean age of 4 months (range between 2 weeks and 24 months). To meet the inclusion criteria in the studies, GERD was diagnosed in all of them through clinical manifestations (using different questionnaires: Modified GERD Symptom Questionnaire in Infants (GSQ-I), i-GERQ, and i-GERQ reviewed (i-GERQ-R)). Three of these RCTs compared PPIs by using a placebo [38,40,41], and another took into account body position and other anti-reflux therapies (Mylanta^®^) as controls [39]. 

Loots et al. [39] found that the number of GER episodes and esophageal acid exposures were significatively lower in infants who received PPIs (esomeprazole) and in the left lateral position (LLP). These results are not correlated with a significant improvement in symptoms, other than in vomiting. Comparing PPIs independently with antacids, PPIs produced a reduction in the reflux index but this was not significant. The same occurred when LLP was compared with the cot head elevation (HE) where LLP produced a considerable reduction in total GER episodes (21 vs. 10, *p* = 0.056). 

In contrast, Winter et al. (2010) [40] found that, compared with the baseline, during the initial 4-week pantoprazole treatment period, all patients experienced a significant improvement in symptoms as analyzed through weekly GERD symptom scores (WGSSs) [*p* < 0.001]. However, during the double-blind phase of the study, no significant differences were found in terms of symptom worsening or improvement between the placebo group and the treatment group. Two years later, the same authors published a study [41] in which, in the open-label (OL) phase, esomeprazole was administered to all patients, resulting in a pronounced improvement in clinical scale and symptom scores. In the double-blind (DB) phase, symptoms worsened in patients who received a placebo and in those who received esomeprazole, without significant differences (48.8% vs. 38.5%, *p* = 0.28).

A significant improvement in symptoms was also observed at the end of the OL phase with rabeprazole (10 mg/day) in the study by Hussain et al. [38]. Again, no significant differences were found in the DB phase between the three groups (patients receiving rabeprazole 5 mg/day, rabeprazole 10 mg/day, or a placebo).

#### 3.2.2. Child Population

Regarding children between 1 and 18 years old with GERD, six articles were identified [36,42,43,44,45,46]. Three of them [44,45,46] studied children aged between 1 and 11 years, one those from 12 to 36 months [43], one between 1 and 5 years old [42], and one [36] studied the adolescent population (12–17 years old). The studies included 453 infants (55.1% males) with a mean age of 5.6 years (ranging between 1 and 17 years old) and GERD was diagnosed through an endoscopic study in all of them. Table 3 summarizes the studies that examined children.

The published articles demonstrate that PPI treatment is effective in achieving endoscopic healing in patients with EE in a high percentage of cases, reaching 100% in the studies published by Tolia et al. [43] and Baker et al. [42]. No significantly higher rates of endoscopic remission were found when using higher doses of PPIs [43,44,45,46]. 

Clinical improvement aligns with histological improvement in most of the studies within this age range. Tolia et al. [43] noted a significant enhancement in symptoms across various treatment doses compared with the baseline. Similar findings were observed by Baker et al. [42] where symptoms improved in all treatment groups, although this was not statistically significant in the group receiving a medium dose (0.6 mg/kg/day of pantoprazole).

An OL phase with dexlansoprazole for 8 weeks was carried out in the study by Gremse et al. [36]: 88% of patients achieved endoscopic healing, entered the maintenance phase, and were randomized into two groups (PPI treatment or a placebo). For patients with Los Angeles grade A EE, similar healing rates were observed between dexlansoprazole [82%] and a placebo [87%]. In cases of grade B, differences with treatment were more pronounced (82% healing with dexlansoprazole and 13% in the placebo group). Only one patient had baseline grade C EE; the patient received a placebo and did not see sustained healing. 

#### 3.2.3. Others

One article [37] involved patients aged 0 to 18 years with symptomatic GERD. In this study published by Zohalinezhad in 2015 [37], patients were randomized into two treatment groups based on whether they received quince syrup or omeprazole. The diagnosis of GERD was made using clinical scales: the GSQ-I was employed for patients aged 1 to 4 years, the GSQ for young children (GSQ-YC), and the GERD assessment of symptoms in Pediatric questionnaire (GASP-Q) for patients aged 5 to 18 years.

The study included 79 patients (mean 67.66 months). The most common symptom was refusal to eat/refusal to feed in young children, and burping or belching in older children. As seen in both younger patients and older children, symptoms and weight significantly improved during and after treatment, but there were no significant differences between the control and treatment groups. 

### 3.3. Safety and Adverse Events

In terms of safety, all but two studies found AEs [35,37]. Among neonates, only Davidson et al. [34] found AEs that were comparable between both groups (23.1% esomeprazole group, 34.6% placebo group). The predominant AE was desaturation (two patients in the esomeprazole group and one in the placebo group).

In infants over 1 month of age, all authors reported AEs [38,39,40,41]. The majority of the reported AEs were not related to treatment with PPIs (no significant difference compared with the placebo group) and were generally mild to moderate. The most frequent AEs were associated with gastrointestinal symptoms (diarrhea, constipation, vomiting), upper respiratory tract infections, and fever/pyrexia. Two of these authors documented serious AEs, including gastroenteritis (one patient with a rotavirus infection), failure to thrive, reduced oral intake, and weight loss [39,40].

Two articles found AEs that were possibly or likely related to treatment administration [38,41]. Winter et al. [41] identified four patients with treatment-related AEs (abdominal pain, tachypnea, regurgitation, and alanine aminotransferase elevation), and two of them persisted in the DB phase, (tachypnea—esomeprazole group, alanine aminotransferase elevation—placebo group). 

In children, AEs were described in all included articles [36,42,43,44,45,46], with no significant differences between PPIs and a placebo or with different doses of PPIs. 

Most of these were mild to moderate. The most common treatment-related AEs were those related to gastrointestinal symptoms and upper respiratory tract infections, rhinitis, and nasopharyngitis. In this group, headaches were also described as being frequent and mild to moderate.

The treatment-related AEs described were diarrhea and sleep disturbance with pantoprazole [42], abdominal pain in one patient, and a decrease in appetite in another one in the dexlansoprazole treatment group [36]. 

Three authors reported serious treatment-emergent adverse events [TEAEs] and these included a case of convulsions (dexlansoprazole), the recurrence of GERD (dexlansoprazole), and H1N1 influenza (placebo) [36]. Haddad et al. [45] reported severe TEAEs: abdominal pain, nausea, vomiting, bronchopneumonia, gastroenteritis, and coughing and choking; they also described lymphadenitis, bronchopneumonia, and seizures [46].

Elevated serum gastrin levels were reported by Hussain et al. [38] (rabeprazole) and Gremse et al. [36]. The mean serum gastrin levels for subjects that were treated with dexlansoprazole in a placebo group later decreased to near-baseline [36]. 

No study disclosed alterations in vital signs. There were no reported deaths in any of the studies during their course.

## 4. Discussion

In our review, we assessed the effectiveness and safety of different PPIs for the treatment of GERD in various age groups. Despite the increasing use of PPIs in the pediatric population, our study did not find significant results in symptom improvement in the RCTs performed in infants. These results are consistent with the previous systematic review by van der Pol [30], in which the use of PPIs was as effective as ranitidine or antacids in reducing GERD symptoms, with no benefit if different doses of the same PPI were used [30]. In children, PPIs are effective in improving symptoms and achieving endoscopic healing, but this is without differences between the various dosage groups. 

The evidence regarding the use of PPIs in the pediatric population for GERD is of very uncertain quality due to various factors, including the diversity of the population, variability in outcome measures, and the limited availability of RCTs comparing responses to PPI treatment with a placebo.

Most of the articles selected in our review, especially regarding infants, used diagnostic clinical scales for GERD as inclusion criteria: Winter et al. (2010) [40] studied patients with a GSQ-I mean symptom frequency > 16; Loots et al. [39] and Hussain et al. [38] employed the I-GERQ-R, and Zohalinezhad et al. [37] used age-specific questionnaires: GSQ-I (infants), GSQ-YC (children 1–4 years), and GASP-Q (5–18 years). The ESPGHAN guidelines [11] stated that GERD diagnosis can usually be carried out without additional complementary testing and there is no clinical tool that serves as the gold standard [3,11]. These scales typically include questions about the frequency and severity of symptoms such as regurgitation, vomiting, irritability, and sleep problems [47,48]. Although reaching a diagnosis of GERD in infants can be challenging, many symptoms present in these scales may also be present in other diseases, and the evidence regarding their use is scarce, evolves over time, and depends on the preference and clinical practice of healthcare professionals, as well as the guidelines and recommendations in place. 

In children older than 12 months, additional tests are often necessary to exclude other diseases. This fact is reflected in our review because all patients included in this age group [36,42,43,44,45,46] underwent an endoscopy as part of the inclusion criteria and to assess the effectiveness. There is no sufficient evidence to support carrying out an endoscopic study to achieve GERD diagnosis [11], but it could be interesting to detect EE, microscopic esophagitis, or complications before escalating treatment [1,11] and in clinical trials to ascertain endoscopic healing with PPIs, as presented here. 

The incidence of EE in the population included in this review was highly variable. Baker et al. [42] reported an incidence of 6.7%, and in the studies by Haddad [45,46] and Tolia [43,44], EE was present in between 39 and 49% of cases. This prevalence is higher than that reported in other studies, possibly because, in some of these studies [43], the patients did not initially respond to other measures. Gilger et al. [49] reported a global incidence of 12.4%, ranging from 5.5% in infants to 19.6% in 17-year-olds. In the included studies [36,43] that assessed the diagnosis and severity of EE using the Los Angeles [LA] classification system [50], the majority of included patients had mild and moderate EE, likely indicating a lessened severity of GERD in children. 

The effectiveness of PPI treatment in the studies included in this review was assessed predominantly through clinical scales, especially in infants. The studies carried out in neonates evaluated the effect of PPIs with complementary tests: pharyngoesophageal motility testing [35] or esophageal pH impedance [34]. Only some studies carried out in children older than 12 months investigated endoscopic remission after treatment with PPIs; however, this was only performed in patients with EE at baseline endoscopy [36,42]. In most of the studies, a baseline biopsy was only performed in doubtful cases to assess histological esophagitis [42]. Baseline biopsies were carried out in all patients included in the study by Tolia et al. [43], but only Haddad et al. [45] included two histological studies (basal and post-treatment) as part of their study and checked histological changes. In the study by Gremse et al. [36], 82% of patients achieved endoscopic healing with dexlansoprazole at week 16, and histological healing was maintained in 76.1% of children treated with rabeprazole at week 24 according to the article by Haddad et al. [46]. In contrast, studies conducted in adults showed that only 66% of patients maintained remission [36], showing that adults often present more severe degrees of EE [36]. In fact, the percentage of maintenance in pediatric studies was lower in patients with moderate-to-severe disease [46].

Although they checked endoscopic remission, the majority of these studies [43,44,45] compared the effectiveness of different doses of the same PPI, without differences between dosage groups. Only Gremse et al. [36] compared dexlansoprazole with placebo with endoscopic criteria, in addition to clinical scales [36] or pH impedance [36]. 

Regarding the treatment period for evaluating effectiveness, it was 8–12 weeks in most groups (ranging from 2 to 24 weeks). Four studies were conducted in two phases [36,38,40,41] where symptoms improved significantly during the OL phase, and this improvement was sustained in a significant percentage of patients during the DB phase across various treatment groups (PPIs, different PPI doses, and placebos). 

Different factors may also play a role in how well the treatment works, especially in neonates and infants. In 2016, Kaguelidou [51] conducted a study to find the smallest amount of omeprazole that effectively treats pathological acid reflux in newborns. The study found that the minimum effective amount tends to be higher in older neonates and those born very prematurely, compared with neonates near to full term. In infants under 1 year of age, the results of DB RCTs, in which PPIs are compared with a placebo, have not found significant benefits, although one study [39] did see an improvement in exposure to acidic content with esomeprazole compared with antacids (*p* = 0.043). In these studies, symptoms improved significantly during the OL phase, but this improvement was also maintained during the DB phase in placebo groups. There remains doubt as to whether the improvement in GERD symptoms is related to treatment or the maturation process during the first months of life when a reduction in episodes of LES relaxation happens, solid foods are introduced, and infants stay incorporated for longer [3,40].

Based on NASPGHAN-ESPGHAN guidelines [11], further studies assessing non-PPI measures (i.e., FM and body position) are needed in infants. These measures have only been evaluated in the study published by Jadcherla et al. [35]. This trial compares a group receiving only PPI treatment with another receiving PPIs along with FM (volume restriction, slow feeding in the right lateral position, and supine postprandial position). The group with PPI and FM did not see improvements in esophageal reflexes, respiratory changes, or symptoms [35]. Surprisingly, no study has been found that evaluates the implementation of a cow’s milk protein exclusion diet versus PPI treatment in infants with suspected GERD.

Regarding AEs, PPIs appear to be safe in the pediatric age group. Episodes of desaturation are most frequent in the neonatal period. From one month of age onward, infants and children more commonly experience upper respiratory tract infections and gastrointestinal disturbances.

Most studies in the field of AEs with PPIs focus on long-term use in the adult population [52,53]. Overall, systematic reviews show that PPIs are safe and well-tolerated drugs when used over a short period of time [54], but prolonged use is associated with a greater risk of respiratory and gastrointestinal infections [55,56]. In our review, the most frequently reported AEs were gastrointestinal disorders (vomiting, abdominal pain, and diarrhea) and upper respiratory infections although these were not directly treatment-related. In infants under 1 year of age, infections of the upper respiratory tract were the most frequent AEs, but doubt arises as to whether these were treatment-related as no significant differences were found with a placebo [34,38,40,41] and respiratory infections of viral origin are very common at this age.

AEs do not seem to be related to the drug dosage administered. Hussain et al. [38] reported that children who received a lower drug dose (6.7% in the group receiving 5 mg of rabeprazole) experienced more AEs than those who received a higher drug dose (2.3% in the group receiving 10 mg of rabeprazole).

Other AEs associated with the prolonged use of PPIs are related to the malabsorption of minerals or nutrients such as magnesium and vitamin B12 [55]. Surprisingly, no studies in our systematic review or published, to the best of our knowledge, evaluate these circumstances [55]. 

It has been described that the prolonged use of PPIs increases the production of gastrin [57,58,59] as a compensatory response of G cells. In our review, two authors reported an increase in serum gastrin levels with rabeprazole [38] or dexlansoprazole [36]. In adults, prolonged hypergastrinemia has been associated with hyperplasia of enteroendocrine cells and gastric carcinoid tumors [60,61]. In children, hypergastrinemia secondary to the use of PPIs does not seem to be a concern as it has been observed that gastrin levels return to normal once the treatment is discontinued for a short period (<12 weeks) [40,55].

The extended use of PPIs could also potentially lead to bone fractures as a result of a decrease in calcium absorption and the inhibition of osteoclasts [55]. Data on adults are controversial. In our review, no episodes of bone fracture were described. Wang et al. [62] conducted a study involving over 100,000 patients under the age of 14. They observed a significantly higher risk of minor fractures with PPI treatment (upper and lower limbs), but no association with spinal or head fractures. The risk of fractures depended on the PPIs used (there was a risk only with omeprazole) and the duration of treatment, but no relation was found with the daily dose [28,62]. Prior to this study, in 2015, Freedberg et al. [22] conducted research in a very extensive patient cohort, and they observed a dose–response effect with increased total exposure to PPIs in young adults (18–29 years old), but not in children (<18 years). In 2019, Malchodi et al. [27] published a study on 851,631 children who received treatment with antacids (PPIs or H2 antagonists) during their first year of life. After adjusting for covariates, they found that patients who received PPIs alone (a 23% higher risk) or in combination with H2 antagonists (a 31% higher risk) had a higher risk of bone fractures. This association was not found in patients who received only H2 antagonists [27,28].

While preparing this manuscript, a systematic review of the pharmacological treatment of GER in children was published by Cochrane [1]. This review differs from our work: all studies with patients with GER (not just GERD) were included, and studies that analyzed non-pharmacological treatments were excluded (i.e., dietary measures). The patients included were under 16 years of age, while, in our study, we included patients up to 18 years of age (as in the study by Zohalinezhad et al. in 2015 [37] and that by Gremse et al. [36]). Finally, the study period lasted until September 2022, not June 2023. 

The limitations identified in the studies and the justifications for conducting a proper systematic review are as follows. First, in daily clinical practice, it is challenging to distinguish between GER and GERD, and these terms are often incorrectly used interchangeably. Second, symptoms are highly variable, nonspecific, and change with age, posing a diagnostic challenge, especially in infants who often present with crying and irritability—symptoms that can be present in various pathologies. Third, due to these obstacles, establishing the true prevalence of GERD is difficult. Fourth, there is currently no clinical tool that serves as the gold standard for diagnosing GERD in the pediatric population. Fifth, the majority of studies conducted in children do not incorporate a placebo control group and instead investigate the response to various doses of the same PPI; hence, it was not possible to study spontaneous healing in these patients.

However, our study summarizes the current evidence and is an initial step for future work looking at pathologies as common in children as GERD. Future possible lines of research could be studies comparing the effectiveness of PPIs with other non-pharmacological measures, such as postural measures, the thickening of intake, or, especially, a cow’s milk protein exclusion diet.

## 5. Conclusions

So far, there is no strong evidence that treatment with PPIs improves symptoms in infants with GERD. In children and adolescents, the use of PPIs was effective in achieving histological remission in patients with EE, accompanied by an improvement in symptoms. 

Despite this, PPIs have been found to be safe and well-tolerated drugs in all age groups when used for a short period of time. It is crucial to prescribe these drugs judiciously, keeping in mind the described side effects, especially when employed over extended periods. More studies in the pediatric population are necessary to investigate the effectiveness and safety of PPIs. 

## Figures and Tables

**Table 1:** PICO criteria [32] for the inclusion of studies on the efficacy and AEs of PPIs for the treatment of GERD.

Inclusion Criteria	
Population	Patients from birth to 18 years old with GERD not secondary to another gastrointestinal pathology
Intervention	The administration of PPIs for the treatment of GERD
Comparison	Another PPI, another dose of PPIs, placebo, no treatment, alternative therapy for GERD (antacid or H2 blocker)
Outcomes	Effectiveness of PPIs: Improvement in clinical symptoms Need for rescue medication Changes in pH impedance Endoscopic and histological remission Adverse events
Study design	Restricted to RCTs

GERD: gastroesophageal reflux disease; PPIs: proton pump inhibitors; RCTs: randomized controlled trials.

**Table 2:** Characteristics of studies carried out in neonates and infants.

Study	Objective, Participants, Diagnosis	Intervention (N, Age)	Control (N, Age)	Results	Adverse Events
NEONATES					
**Davidson et al. (2013) [34]**	Esomeprazole vs. placebo. Neonates (PMA 28–44 w); clinical findings reproducible (8 h video-cardio-respiratory monitoring)	Esomeprazole 0.5 mg/kg/day (*n* = 25, 48.1 +/− 29.8 days)	Placebo (*n* = 26, 46.5 +/− 31.2 days)	No statistically significant difference in the total number of GERD-related signs and symptoms (esomeprazole: 14.7%, placebo: 14.1%, *p* = 0.92)	Esomeprazole: 23.1%, placebo: 34.6%. Most commonly reported: gastrointestinal disorders (9.6%), desaturation (2 esomeprazole, 1 placebo)
**Jadcherla et al. (2020) [35]**	Esophageal induced reflexes Neonates (PMA 36–40) Symptoms and pH impedance ARI ≥ 3%	Omeprazole 0.75 mg/kg/dose + FM bundle (*n* = 25, PMA 41.2 +/− 3.1 w)	Omeprazole 0.75 mg/kg/dose (*n* = 24, PMA 41.4 +/− 2.2 w)	No different peristaltic reflex (OR = 0.8, 95% CI 0.4–1.6, *p* > 0.99) Follow-up: distal esophageal contraction and LES tone decreased LES relaxation reflex less frequently (*p* < 0.05)	No AEs found in the study
INFANTS					
**Winter et al. (2010) [40]**	Efficacy of pantoprazole; infants (1 and 11 m) Modified total GSQ-I > 16 and a clinical diagnosis of suspected, symptomatic, or endoscopically proven GERD	Pantoprazole 5 mg/day for infants 2.5 kg to <7 kg 10 mg/day for infants >7 kg to 15 kg (*n* = 52, 5.15 +/− 2.81 m)	Placebo (*n* = 54, 5.04 +/− 2.81 m)	OL phase: significant reduction in WGSSs from baseline (*p* < 0.001) with pantoprazole DB phase: the decrease continued for both treatment groups; no significant differences in withdrawal rates due to lack of effectiveness	AEs recorded: 29 in pantoprazole group, 19 in placebo group (no significant differences, all mild or moderate). Most common AEs in both groups: upper respiratory tract infections (13%)
**Winter et al. (2012) [41]**	Efficacy and safety of esomeprazole; infants (1 to 11 m); GERD diagnosed by symptoms, confirmed by endoscopy or an investigator’s determination of GERD	Esomeprazole 2.5 mg/day (3–5 kg) 5 mg/day (>5–7.5 kg) 10 mg/day (>7.5–12 kg) (*n* = 39, 4.9 +/− 2.6 m)	Placebo (*n* = 41, 4.9 +/− 3.2 m)	OL phase: 82.7% symptom improvement DB phase: no significant differences between the treatment group and the placebo group regarding symptom worsening (38.8% vs. 48.5%, HR 0.69; 95% CI 0.35–1.35%; *p* = 0.28)	OL phase: 48% of patients AEs DB phase: 59% esomeprazole, 66% placebo Most common: upper respiratory tract infection (15.4% and 9.8%, respectively). No serious treatment-related AEs considered
**Hussain et al. (2014) [38]**	Efficacy and safety of rabeprazole, infants (1 to 11 m), GERD resistant to conservative therapy and/or previous acid-suppressive medications, I-GERQ >16	Rabeprazole (*n* = 178, 4.7 +/− 2.54) Rabeprazole 5 mg (*n* = 90, 4.6 +/− 2.57) Rabeprazole 10 mg (*n* = 88, 4.7 +/− 2.52 m)	Placebo (*n* = 90, 4.7 +/− 2.65 m)	No differences in primary efficacy variables Frequency of regurgitation (−0.79 vs. −1.20 times/day; *p* = 0.16) Mean increase weight—z scores (0.11 (0.329) vs. 0.14 (0.295); *p* = 0.440) I-GERQ score (−3.6 (−25%) vs. −3.9 points (−27%); *p* = 0.960)	Similar rates of AEs (47%) both in the placebo and combined rabeprazole groups Most common AEs: pyrexia (2% placebo, 7% rabeprazole), upper respiratory tract infection (6% vs. 5%), GERD (8% vs. 4%), and vomiting (6% vs. 3%)
**Loots et al.** **(2014) [39]**	Efficacy of LLP in GERD Infants (birth–6 m). pH impedance, monitoring, 8 h video study, gastric emptying breath test, I-GERQ q	Group 1 LLP + ES 1 mg/kg/day (*n* = 12, 12 +/− 3 w) Group 2 HE + ES 1 mg/kg/day (*n* = 14, 12 +/− 3 w)	Group 3 LLP + AA (*n* = 13, 14 +/− 2 w) Group 4 HE + AA (*n* = 12, 17 +/− 2 w)	Vomiting was reduced in AA + LLP (*p* = 0.042). LLP compared with HE produced a greater reduction in total GER (*p* = 0.056) Acid exposure was reduced with PPI compared with AA (*p* = 0.043)	No AEs correlated with treatment Five patients experienced AEs (urinary tract infection, constipation, diarrhea, vomiting)

Abbreviations: AA: antacid; AEs: adverse events; ARI: acid reflux index; DB: double-blind; FM: feeding modifications; ES: esomeprazole; GERD: gastroesophageal reflux disease; GSQ-I GERD Symptom Questionnaire in Infants; HE: head of cot elevation; HR: hazard ratio; I-GERQ: infant gastroesophageal reflux questionnaire; LES: lower esophageal sphincter; LLP: left lateral position; m: months; OL: open-label; PMA: postmenstrual age; w: weeks; WGSSs: weekly GERD symptom scores.

**Table 3:** Characteristics of studies carried out in children.

Study	Objective, Participants, Diagnosis	Intervention [N, Age]	Control [N, Age]	Results	Adverse Events
**Tolia et al. (2010)** [44]	Endoscopic healing of EE Children 1–11 y. Endoscopically confirmed GERD	Esomeprazole (8 w) <20 kg: 5 mg (*n* = 26, mean 2.1 y, EE = 12). ≥20 kg: 10 mg (*n* = 31, mean 8.5 y, EE = 16)	Esomeprazole <20 kg: 10 mg (*n* = 23, mean 2.5 y, EE = 12) ≥20 kg: 20 mg (*n* = 29, mean 8.3 y, EE = 13)	109 randomized patients: 49% EE EE healed in 89%: <20 kg/5 mg 100%, <20 kg/10 mg 82%, ≥20 kg/10 mg 90%, ≥20 kg/20 mg 85%	10/108 patients with AEs related to esomeprazole [9.3%] 13 AEs reported Most common: diarrhea [*n* = 3], headache [*n* = 2], somnolence [*n* = 2]
**Tolia et al. (2010)** [43]	EE healing and symptom improvement Children 12–36 m with GERD Diagnosis by clinic (PGA) and endoscopy	Esomeprazole (8 w) 5 mg (*n* = 18, mean 21.8 m) 10 (56%) EE (28% LA grade A, 28% LA grade B) Baseline PGA: 45% mild, 50% moderate, 5% severe	Esomeprazole (8 w) 10 mg (*n* = 13, mean 22.5 months) 5 (39%) EE (23% LA grade A, 8% LA grade B, 8% LA grade C) Baseline PGA: 20% mild, 80% moderate	31 patients: EE: 15 (48.4%), control: 100% healed Final PGA: 5 mg: 45% none, 50% mild, 5% moderate 10 mg: 20% none, 65% mild, 15% moderate	Most common AEs: vomiting, pyrexia, and diarrhea
**Baker et al. (2010)** [42]	GERD symptom improvement Children 1–5 years: GSQ-YC >3 and endoscopic HE (Hetzel Dent grade ≤ 2) or EE (Hetzel Dent grade > 2)	Pantoprazole (8 w) 0.3 mg/kg (LD): *n* = 18, 2.7 years (+/−1.6)	Pantoprazole (8 w): 0.6 mg/kg (MD): *n* = 21, 1.9 y (+/−1.2) 1.2 mg/kg (HD): *n* = 21, 2.8 y (+/−1.3)	60 patients (56 HE, 4 EE) Improvement in WGSS (HE population, 8 w): LD: *p* < 0.001, MD: *p* = 0.063, HD: *p* < 0.001 Endoscopic healing: 100% of the EE population	Most common AEs: upper respiratory infection, fever, diarrhea, rhinitis, vomiting, headache
**Haddad et al. (2013)** [45]	Endoscopic healing at 12 w Children 1–11 years old endoscopically/histologically GERD (Hetzel-Dent ≥1 and Histological Features of Reflux Esophagitis scale >0) and at least one symptom of GERD	Rabeprazole (12 w) <15 kg (LW): 5 mg (*n* = 21, 2.4 +/− 1.2 years, H-D score 1.7 +/− 0.97) ≥15 kg (HW): 10 mg (*n* = 44, 7.6 +/− 2.9 years, H-D score 1.5 +/− 0.70)	Rabeprazole (12 w) <15 kg (LW): 10 mg (*n* = 19, 1.9 +/− 1.1 years, H-D score 1.4 +/− 0.60) ≥15 kg (HW): 20 mg (*n* = 43, 7.0 +/− 0.7 years, H-D score 1.4 +/− 0.62)	108 patients: 87 endoscopic healing LW/5 mg: 82%, LW/10 mg: 94% HW/10 mg: 76%, HW/20 mg: 78% Change in GERD symptoms severity scores: LW/5 mg: −13.6, LW/5 mg: −9 HW/10 mg: −10.6, HW/20 mg: −8.3	76% of children experienced at least 1 AE, 5% a serious AE Cough (14%), vomiting (14%), abdominal pain (12%), diarrhea (11%)
**Haddad et al. (2014)** [46]	Endoscopic healing at 24 w. Children 1–11 years old with endoscopic healing at 12 w in previous study (Hetzel-Dent 0 and Histological Features of Reflux Esophagitis scale = 0)	Rabeprazole (12 w) <15 kg (LW): 5 mg (*n* = 9, 2.4 +/− 1.24 years) ≥ 15 kg (HW): 10 mg (*n* = 24, 7.7 +/− 2.74 years)	Rabeprazole (12 w) <15 kg (LW): 10 mg (*n* = 8, 1.5 +/− 0.53 years) ≥ 15 kg (HW): 20 mg (*n* = 23, 7.2 +/− 2.66 years)	52 patients, 47 (90%) endoscopic healing LW/5 mg: 100%, LW/10 mg: 100% HW/10 mg: 89%, HW/20 mg: 85% Change in GERD symptoms severity scores: LW/5 mg: −3.8, LW/5 mg: −3.6 HW/10 mg: −2.6, HW/20 mg: −3.0	63% at least 1 AE (5% severe). Upper respiratory tract infection (63%), vomiting (11%), abdominal pain (8%), diarrhea (6%), 5% related to medication
**Gremse et al. (2018)** [36]	Treatment of emergent adverse events and healing of EE Adolescents 12–17 years old with symptoms and endoscopically confirmed EE and healing with dexlansoprazole 60 mg /day (8 w)	Dexlansoprazole 30 mg 16 w treatment period (*n* = 22, 14.6 +/− 1.41 years, LA grade A 61.5%, grade B 34.6%, grade C 3.8%)	Placebo 16 w treatment period (*n* = 24, 14.8 +/− 1.75 years, LA grade A 56.0%, grade B 44%, grade C 0%)	62 patients, 16-week treatment period Healing: dexlansoprazole 82%: grade A 82%, grade B 82% Placebo 58%: grade A 87%, grade B 13%	72.0% (D), 61.5% (placebo) More common: headache (24.0% D, 15.4% placebo) ≥5% D: abdominal pain, nasopharyngitis, sinusitis, upper respiratory tract infection

Abbreviations: AEs: adverse events; GERD: gastroesophageal reflux disease, EE: erosive esophagitis; PGA: Physician Global Assessment; w: weeks of treatment, LA: Los Angeles classification for erosive esophagitis, HE: histologic esophagitis, WGSS: weekly GERD symptom score, GSQ-YC: GERD Symptom Questionnaire for Young Children, LW: low-weight cohort, HW: high-weight cohort, D: dexlansoprazole, y: years.All articles included in this group compared different doses of PPIs with the aim of achieving remission [43,44,45,46] and improving symptoms [42]. In contrast, Gremse et al. [36], after an OL phase with dexlansoprazole treatment, compared the drug with a placebo in terms of histological remission and safety.

## Data Availability

The data presented in this study are available in article and Supplementary Materials.

## Supplementary Materials

The following supporting information can be downloaded at: https://www.mdpi.com/article/10.3390/children11030296/s1. Table S1: Full-text articles assessed for eligibility that were read in full but not included in the review.
