# Supplementary material for: Proton Pump Inhibitors in Pediatric Gastroesophageal Reflux Disease: A Systematic Review of Randomized Controlled Trials
Source: Children (Basel). 2024 Mar 1;11(3):296. doi: 10.3390/children11030296 (PMC10969042; doi:10.3390/children11030296)
Supplement: Supplementary file 1 [file children-11-00296-s001.zip › children-2870691-supplementary.pdf]

Suplemmentary Table S1. Full-text articles assessed for eligibility but not included in the review

.

Reference	Type Study	Aims/Intervention	Age	Reason of exclusion
**Bestebreurtje P et al. 2020**	Clinical trial	To study the efficacy and pharmacokinetics of rectally administered omeprazole in infants with gastroesophageal reflux disease due to esophageal atresia or congenital diaphragmatic hernia and compare these with orally administered omeprazole	Children	Children with a pathology other than GERD
**Chiang HH et al. 2019**	Randomized Clinical Trial	To compare the clinical efficacy of single doses of dexlansoprazole [modified-release 60 mg] and esomeprazole [40 mg] after 24-week follow-up in patients with mild erosive esophagitis	Adults	Population
**Johnson DA et al. et al. 2016**	Clinical Trial	To identify clinical factors related to resolution of reflux-related sleep disturbance in subjects treated with esomeprazole 20 mg for 14 days	Adults	Population
**Steingoetter A et al 2015**	Randomized Clinical Trial	To assess the effect of proton pump inhibitor therapy on the volume, distribution and acidity of gastric secretions in GERD and healthy subjects	Adults	Population Outcome
**Hatlebakk JG et al. 2016**	Clinical Trial	To compare the ability of laparoscopic antireflux surgery and esomeprazole to control esophageal acid exposure, over a 5-year period, in patients with chronic gastroesophageal reflux disease	Adults	Population
**Walker D et al. 2015**	Randomized Clinical Trial	To assess the clinical superiority of Zegerid versus Losec for rapid relief of heartburn associated with gastro-esophageal reflux disease	Adults	Population

Reference	Type Study	Aims/Intervention	Age	Reason of exclusion
**Moraes-Filho JP et al. 2014**	Clinical Trial	To compare the efficacy of pantoprazole-Mg and esomeprazole in GERD	Adults	Population
**Peura DA et al. 2013**	Randomized Clinical Trial	To determine the impact of PPI therapy on heartburn and regurgitation severity in patients with either non-erosive GERD or erosive oesophagitis	Adults	Population
**Xiao YL et al. 2013**	Randomized Clinical Trial	To investigate whether hydrotalcite was comparable to esomeprazole, a proton pump inhibitor, in on-demand therapy for non-erosive reflux disease	Adults	Population
**Flook NW et al. 2013**	Randomized Clinical Trial	To evaluate the efficacy of esomeprazole in primary-care treatment of patients with unexplained chest pain stratified for frequency of reflux/regurgitation symptoms.	Adults	Population
**Pouchain D et al. 2012**	Randomized Clinical Trial	To compare short-term efficacy of an alginate [Gaviscon®, 4 × 10 mL/day] and omeprazole [20 mg/day] on GERD symptoms in general practice	Adults	Population
**Tan VP et al. 2011**	Randomized Clinical Trial	To study esomeprazole for the treatment of non-erosive reflux disease in Chinese patients	Adults	Population
**Dabholkar AH et al.**	Randomized open-label study	To assess the 12-month safety of dexlansoprazole MR in patients with symptomatic gastro-oesophageal reflux disease	Adults	Population
**Johnson D et al. 2010**	Randomized Clinical Trial	To evaluate the efficacy of esomeprazole on GERD-related nighttime heartburn and associated sleep disturbances.	Adults	Population
